# The mystery of the “air-dried chaplain” solved: the life and “afterlife” of an unusual human mummy from eighteenth century Austria

**DOI:** 10.3389/fmed.2025.1560050

**Published:** 2025-05-02

**Authors:** Andreas G. Nerlich, Peter Hofer, Stephanie Panzer, Christine Lehn, Judith Wimmer, Oskar Nowak, Frank Musshoff, Oliver K. Peschel

**Affiliations:** ^1^Institute of Legal Medicine, Ludwig-Maximilians University Munich, Munich, Germany; ^2^Institute of Legal Medicine, Medical University Graz, Graz, Austria; ^3^Diagnostic and Research Institute of Forensic Medicine, Medical University Graz, Graz, Austria; ^4^Department of Radiology, Paracelsus Medical University, Salzburg, Austria; ^5^Department of Art and Heritage Conservation, Diocese Linz, Linz, Austria; ^6^Institute of Human Biology and Evolution, Faculty of Biology, Adam Mickiewicz University, Poznań, Poland; ^7^Forensic Toxicological Centre (FTC), Munich, Germany

**Keywords:** mummy, internal embalming, tuberculosis, eighteenth century life, paleoradiology, paleohistology, paleotoxicology

## Abstract

The multidisciplinary study of the well preserved cadaver of the so-called “air-dried chaplain” from the church crypt of St. Thomas am Blasenstein (Upper Austria) not only solved the “mystery” of the excellent preservation of the trunk of this unusual mummified human body, but also provided circumstantial information about this historic individual, his life and disease history, and conclusion of his cause of death. The mummy of a 35–45 year old male, radiocarbon dated to the period 1730–1780 CE is most likely that of the local aristocratic parish vicar Franz Xaver Sidler von Rosenegg who had been temporarily delegated to St. Thomas parish from his mother monastery of Waldhausen im Strudengau (Upper Austria). He had a high-quality diet based on terrestrial animal products, showed no signs of major physical work load, was most likely a pipe smoker and suffered from chronic active pulmonary tuberculosis with peripheral and central (hilar) calcifications (primary tuberculous complex) and a right lower lobe cavity with focal heterotopic ossification and potential active inflammation. This latter may have caused acute pulmonary hemorrhage which may have been the cause of death. Most surprisingly, we detected, in the otherwise completely intact abdominal (and pelvic) cavity, extensive packing with foreign material which was identified as a mixture of wood chips, fragmented twigs, large amounts of fabric of various types including elaborate embroidered linen, and even pieces of silk. Furthermore, this embalming method seems to have included high level zinc-ion solution impregnation (most likely zinc-chloride with small amounts of arsenic) and the addition of copper. The packing was inserted into the abdominal body cavity through the rectum. It led to an excellent state of conservation of the trunk, while the face (and skull) and peripheral extremities were less well preserved.

## 1 Introduction

The preservation of human remains offers a unique insight into the life, disease and eventually cause of death of individuals from the past (1–4). Mummified remains provide significantly more information than skeletons and/or teeth. Mummies can be created naturally (5), but occasionally also intentionally, with often very different levels of preservation. While some of the technical aspects of intentional embalming in ancient high cultures, such as in ancient Egypt, are partly understood (6), very little is known about deliberate conservation in European mummies in many periods (4).

In 2003 the doyen of paleopathology, Arthur C. Aufderheide, described in his hand book *The Scientific Study of Mummies* (4) the locally nick-named “air-dried chaplain”, an adult human mummy in the church crypt of the small parish of St. Thomas am Blasenstein, Upper Austria, as a uniquely mummified body of a presumed eighteenth century local clergy man. He assumed this was an example of chance mummification of a body naturally due to the climatic conditions of the crypt where it had been stored for a long time. In his list of various internationally studied mummified human remains, he cited the previous observations by the German-Venezuelan pathologist Ekkehard Kleiss, who was the first scientist to describe this unusually well-preserved body several decades before Aufderheide's report (7). Both scientists confirmed the unusualness of the corpse's preservation (4, 7).

Since then, several local researchers discussed the method of the mummification process raising a whole series of hypotheses that included; external treatment with chemical substances, effects of locally enhanced radioactive radiation, local climatic conditions with constant ventilation, influence of humic acid coming from an oak-coffin, and several others (8, 9) However the method has remained a mystery. At the same time the mummy became more and more a local attraction; isolated miracles associated with the dried body have occasionally been reported (8, 10).

Recent renovation of the crypt offered the unique opportunity to perform a state-of-the-art scientific analysis of the corpse. The resulting study was able to confirm the identity of the person's mummified corpse, and also discovered the way the body had been preserved so well over a long period.

## 2 Materials and methods

### 2.1 The mummy and its history

**Figure 1:**
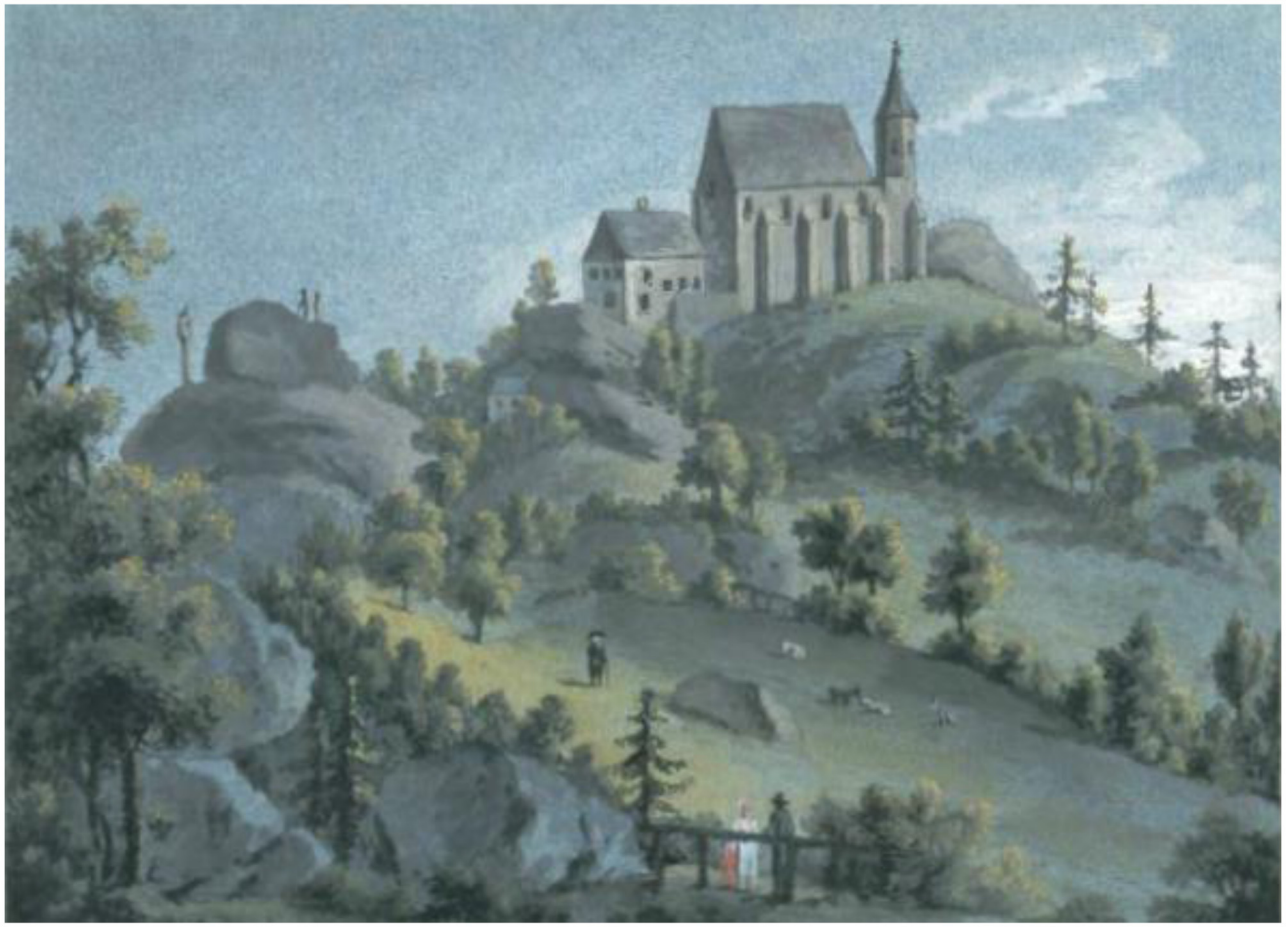
The church of St. Thomas am Blasenstein, Upper Austria, to the right on the hilltop of the so-called “Blasenstein” (right side of the image; literally translated as “bladder stone”) (ca. 1820, unknown artist, collection A. Nerlich).

The mummy of the so-called “air-dried chaplain” is located in the crypt of the church of St. Thomas am Blasenstein, a small village in Upper Austria, a few kilometers north of the river Danube (Figure 1). Rumors suggested that he was a local aristocrat who served as a monk in the nearby Augustinian monastery of Waldhausen im Strudengau (Upper Austria, about 15 km in distance from St. Thomas') and who had been sent as a parish Vicar around 1740. He was called Franz Xaver Sidler (or Sydler) von Rosenegg whose death had been certified in St. Thomas in 1746 (8–12).

Some rumors further suggested that Franz Xaver Sidler had been buried at the local cemetery immediately after death due to an infectious disease. It was said that the well-preserved body had been recovered from the tomb several years later, transferred into the church crypt where several healing miracles occurred associated with this burial (8). The first documented literary evidence of the mummy dates back to 1850 (11). This report, and several subsequent ones, however, does not mention Franz Xaver Sidler at all. Since then the presence of the mummy has been reported repeatedly up to the 1960s however, without further details as to the status of the body, including the preservation status (7).

The first available image of the mummy dates back to 1957. Ten years later Ekkehard Kleiss made a scientific inspection (7) and provided the first detailed description of the body. This, however, covered only the external aspect of the mummy; no measurements were performed or any further technical investigations.

**Figure 2:**
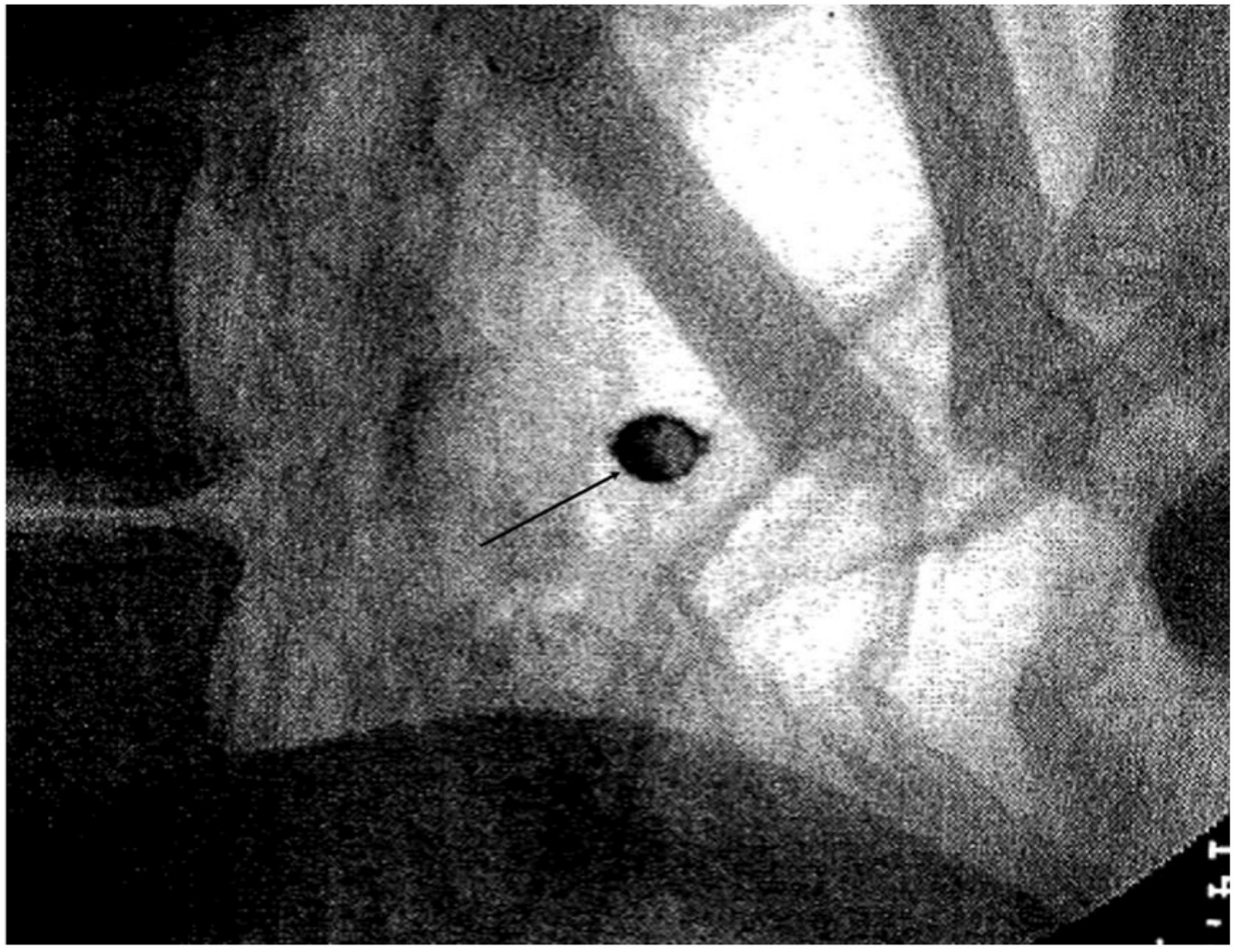
X-Ray from the left lower bowel showing an about 1 cm sized radiodense round structure inside the mummy's abdomen (arrow) raising the suspicion of a poisonous capsule that had been ingested prior to death [X-ray by Dr. F.X. Mayer, courtesy Msgr. Karl Wögerer, Kirchenstiftung Waldhausen/Strudengau, with permission].

In 2000 the body was analyzed in depth by the Austrian pharmacologist Bernhard X. Mayer from the University of Vienna. He performed a detailed external inspection including anthropometric measurements, an X-ray analysis with a portable X-ray machine [B. Mayer, unpubl. results] and took samples for the biochemical analysis of the lipid composition of the subcutaneous fat tissue (13). During his radiographic investigations, he detected a round radiodense “bullet” in the left lower bowel (Figure 2). Since both the ventral and the dorsal body walls were completely intact, this finding increased the speculation that it was a capsule containing poison that may have caused the individual's death (8, 9, 12). These rumors persisted.

For the new examination, the mummy was formally transported to Munich for an extensive interdisciplinary investigation. This had been approved by both the local parish committee and the diocese. The study, besides an archival review, focussed on macro- and micropathological analyses of the cadaver by external inspection, a whole-body CT scan, focal autopsy, contact radiographs of inner organs, radiocarbon and stable isotope analyses, histological, toxicological and forensic investigation and material analyses.

### 2.2 Macroscopical and CT-scan investigation

First, the mummy was inspected macroscopically; its length, body proportions and weight were determined. The body surface and the extent of obvious *post mortem* destruction were recorded. Subsequently, a CT scan of the whole body was performed according to established protocols as previously described in detail [Siemens Somatom, 120V, slice thickness 0.75 cm; (14)].

### 2.3 Focal autopsy and sampling procedure

Following the CT-scan, in order to obtain access to specific internal organs and several unclear items, a focal opening of the chest was necessary (see below). This procedure was performed via a paravertebral longitudinal incision of the dorsal chest wall as previously successfully performed in another mummy study (15). The organs of the chest and abdomen were temporarily removed for contact radiography and sampling for subsequent macroscopic and histological examinations. Also, a bone sample from the vertebral column was taken for stable isotope analysis and skin and subcutaneous tissue was taken for radiocarbon determination and toxicological studies. Finally, a first molar was extracted, also for stable isotope analysis.

### 2.4 Radiocarbon and stable isotope investigation and histological analysis

For stable isotope analyses, collagen was extracted from the dentin, vertebral bone and skin and subcutaneous tissue. Stable isotope analyses of carbon, nitrogen and sulfur were performed on the collagen samples following the usual protocols (16). The selected tissues provided insights into diet and living conditions of the individual during childhood (dentin), the last 15–20 years (bone) and the last few months (skin and subcutis) (16).

The radiocarbon dating on the skin and subcutaneous tissue was performed at the Leibniz-Laboratory for Radiometric Dating and Isotope Research, Kiel University (Germany) using an accelerator mass spectrometry (AMS Type HVE 3MV Tandetron 4130) (17) and calibration data by Reimers et al. (18).

For the preparation of the histological analyses, the tissue samples of skin and subcutis, bone and lung were rehydrated, fixed, bone and some of the lung samples were decalcified. All samples were then embedded into paraffin wax (19, 20).

### 2.5 Toxicological analysis

For this analysis a soft tissue sample was obtained from the dorsal abdominal musculature that was not in direct contact with the skin surface in order to exclude any direct contamination from the surface. The sample was prepared and investigated via multi-element analysis through ICP-MS as routinely applied (21). The resulting pattern covered 24 elements and provided quantitative data which were compared to modern-day reference values (22–24).

In addition, a further part of the sample was also investigated by fluid gas chromatography coupled with mass spectrometry (GC-MS) for the identification of biologically relevant toxicological agents (25).

### 2.6 Material analysis

Since the opening of the dorsal chest wall following the CT-analysis provided access to the interior of the abdomen, the type and composition of the foreign material found was investigated in detail. This was performed with the kind help of the Bavarian Criminal Police Department, München, who provided several important findings based on macro- and microscopical analyses (reflected light microscopy). Furthermore, surface infrared spectrum scanning microscopy [Fourier-transform infrared spectroscopy, FITR (26)] particularly of the previously mentioned round radio-dense foreign body was performed that had also been retrieved from the mummy's abdominal cave.

## 3 Results

### 3.1 The mummy—macroscopic and anthropological findings

**Figure 3:**
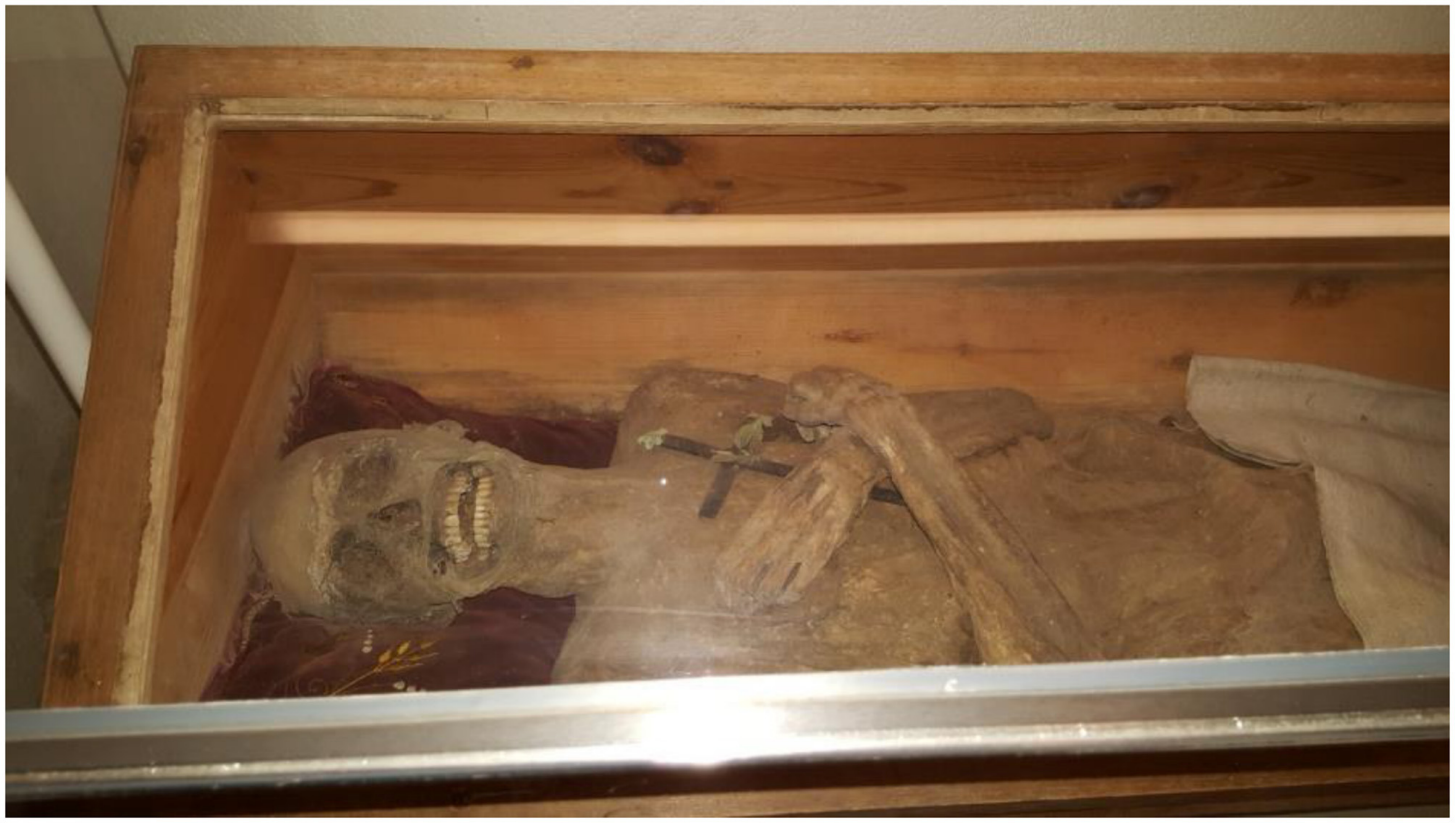
The mummy of the “air-dried chaplain” in his coffin in the church crypt of St. Thomas am Blasenstein, Austria (image: J. Wimmer).

**Figure 4:**
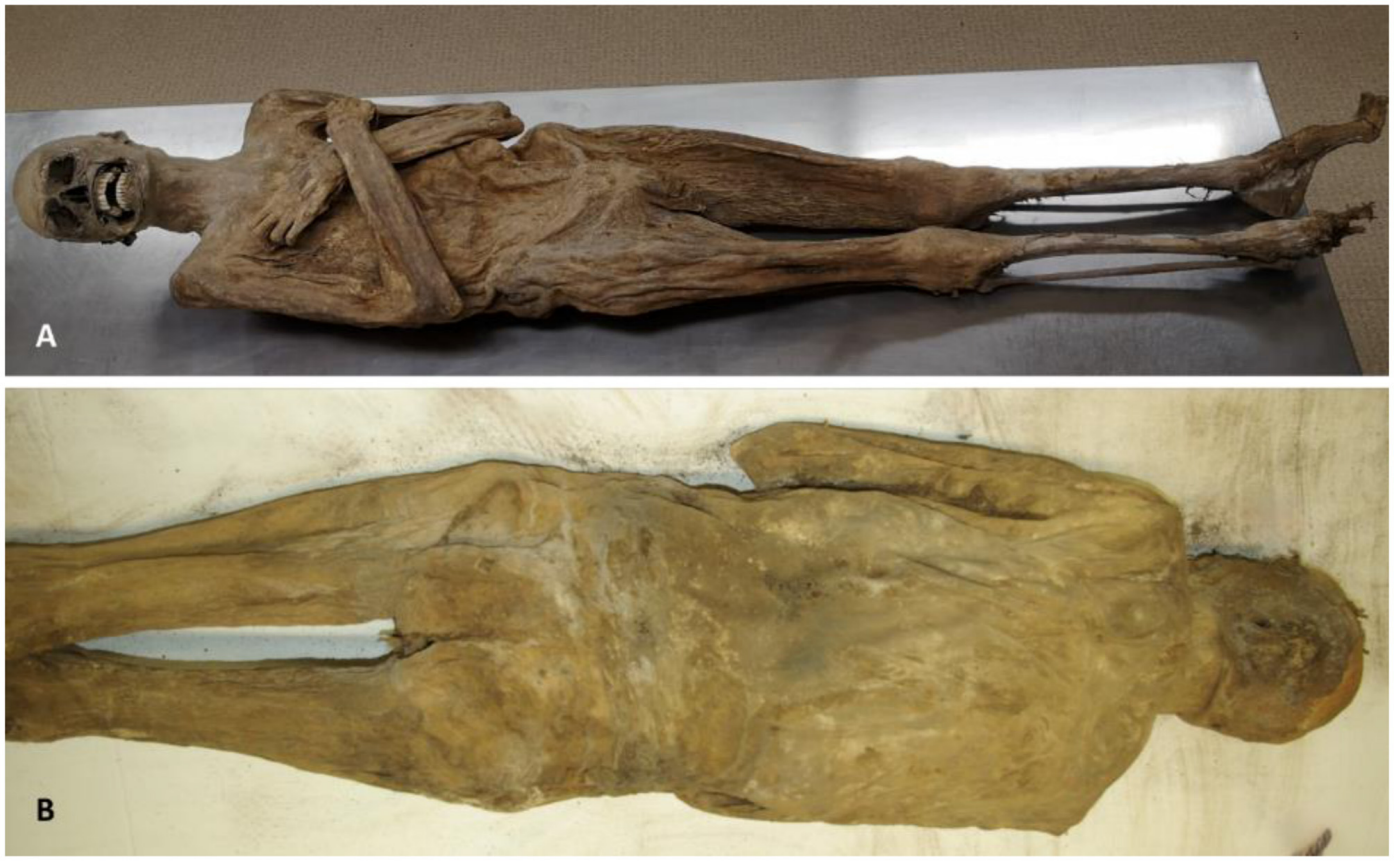
External appearance of the mummy from the ventral **(A)** and dorsal side **(B)** showing a completely intact body wall (images: A. Nerlich).

The body lay completely undressed in its showcase with both arms crossed over the chest (Figure 3). This was an adult male with excellent preservation of the (ventral and dorsal) chest and abdominal walls, both of which were completely intact (Figure 4) with moderately well preserved proximal and distal upper and proximal lower extremities and neck. In contrast, the face and the distal lower extremities show considerable signs for *post mortem* decay, and some parts of the right forefoot were missing (Figure 4).

There was no remaining hair either on the skull or at the typical postcranial body regions such as the axilla or pubic regions. The individual's sex was clearly defined by male external genitalia. Below the axillae, and occasionally on the mummy's backside, there existed minor residual fragments of cloth attached to the body. These disintegrated rapidly and could not further be investigated. Around the waist the body appeared slightly constricted as if surrounded by a belt that had been removed after mummification. Careful inspection of the anal orifice showed slight dilatation, consistent with a *post mortem* plug that may have been used to avoid any leakage of body fluid after death. Any plug and any clothing covering the body might have been removed after death.

**Figure 5:**
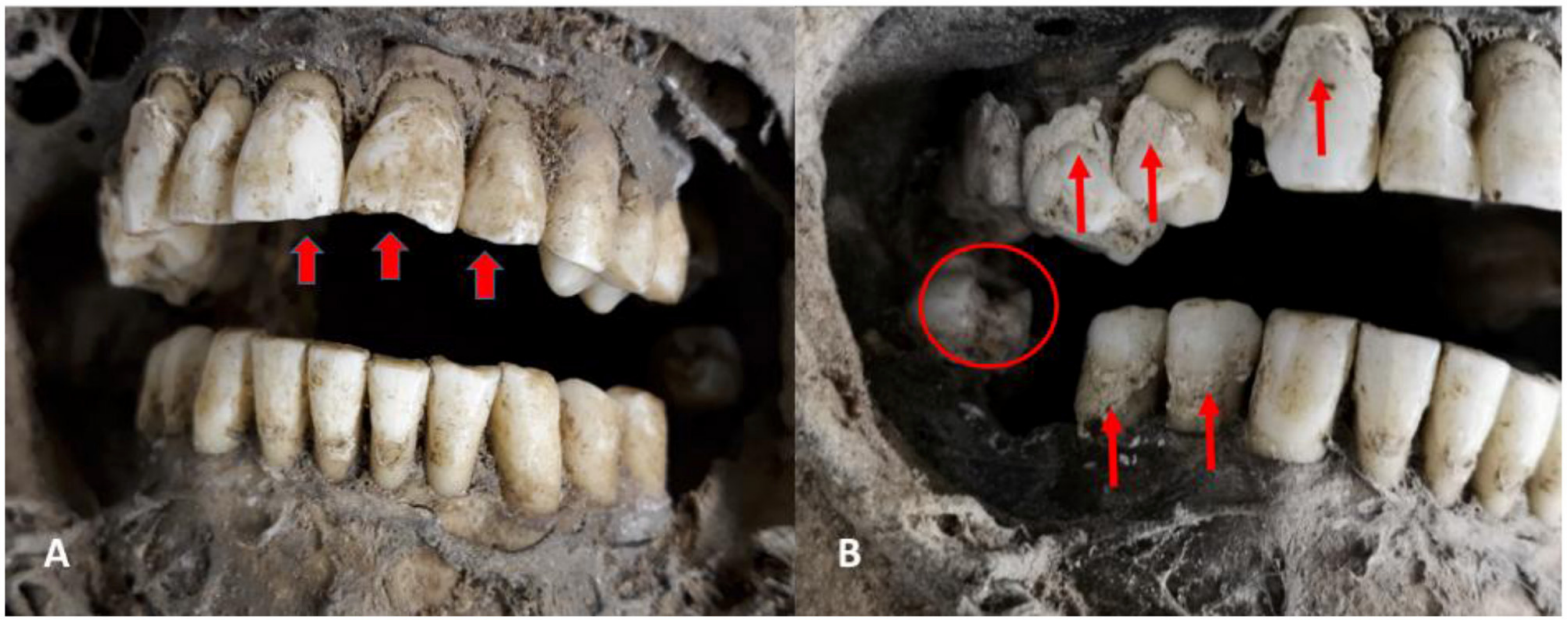
Dental status: **(A)** Frontal view of maxillary and mandibular dentition showing very well preserved and only slightly abraded teeth with slight retraction of the chewing surface on upper incisors (arrows). **(B)** The right lateral part of the dentition additionally revealed retraction of the alveolar sockets (due to periodontitis, no sign), significant dental calculus (arrows) and a dental caries in the tooth of region 48 (circle) (images: A. Nerlich).

The appearance of the face was unusual in that the lips were significantly retracted exposing both the maxillary and mandibular rows of white teeth with some minor evidence for mild dental abrasion, but otherwise excellent preservation. Interestingly, the teeth of region 21 (incisor right maxilla), 31 and 32 (incisors left maxilla) showed a slightly curved retraction of the chewing surface such as seen in individuals who frequently chew on an object such as a pipe (Figure 5A). The tooth of region 48 (right last molar of the mandible) showed significant dental caries and the teeth of the regions 16, 17, 46, and 47 were lost a significant time before death since all the dental alveoli were closed. Finally, several teeth showed signs of periodontal inflammation with retraction of the corresponding dental alveoli, and several teeth were affected by typical dental calculus (tartar) (Figure 5B).

### 3.2 Anthropological evaluation

The mummy weighed 10.5 kg with an overall body length of 170.8 cm in its shrunken state. In order to estimate its intravital body length, several long bones were measured from CT scan images (see below). The bone length of the femora were 474.1 and 469.0 mm (right/left), of the tibiae of 410.1 and 409.2 mm and the humeri of 368.0 and 351.8 mm. Taking Middle European reference values into account a whole body length between 171 and 183 cm was calculated resulting in a mean body length of 176 +/– 2 cm (27). Accordingly, *post mortem* shrinkage of ~5 cm length was concluded.

The evaluation of the individual's age at death was performed on the basis of several criteria. Since the skin was absent from most of the surface of the calvarium, the pattern of closure of cranial sutures could be determined. This showed persistently open sutures over the complete calvarium with some fusion of the high sagittal suture (region S3) suggesting an age at death of between 30 and 50 years (28, 29). The CT scans of the symphysis demonstrated a surface morphology with still quite prominent ridges and grooves. Taking the most recent ranking systems into account (six phases) (30) the following relevant criteria could be considered: the symphyseal face still showed the typical ridge and furrow system, the pubic tubercle was separated from the symphyseal face which had a distinct rim; on the ventral side, there were some inferior outgrowths, and slight lipping on the dorsal border. On the basis of the classification (30) a phase IV was suggested which indicates an individual age of 35.2 +/− 9.7 (1SD) and a 95%-range between 23 and 57 years.

Finally, the spongy bone structure of long bones was estimated (31) which did not show any major rarefication (stage 2) which covered a control age group of 44.0 +/− 10.4 (1SD) and an age range between 19 and 61 years.

### 3.3 CT scanning results

The CT scan of the whole body involved ~2,500 consecutive slices of 0.75 mm thickness and provided insight into all the body cavities/organ systems. In general, all osseous structures of the mummy were in regular shape, there were minimal signs of degeneration in the ilio-sacral joints, minimal osteoarthritis of the right proximal femoral joint (focal mild subchondral osteosclerosis), but no evidence for osteoarthritis in any further weight-bearing large nor small joints nor of the intervertebral junctions. The bone itself is very well mineralised.

Looking at the internal structures from cranial to caudal, the skull cavity contained fragmented remains of brain substance, which was shrunken and dislocated to the dorsal skull cavity along with remnants of the meninges [not shown]. The paranasal sinuses showed slightly thickened walls such as in minor chronic inflammation. The left big toe presented with medial deviation of the metatarso-interphalangeal joint as seen in a mild hallux valgus malformation as a result of wearing narrow shoes.

**Figure 6:**
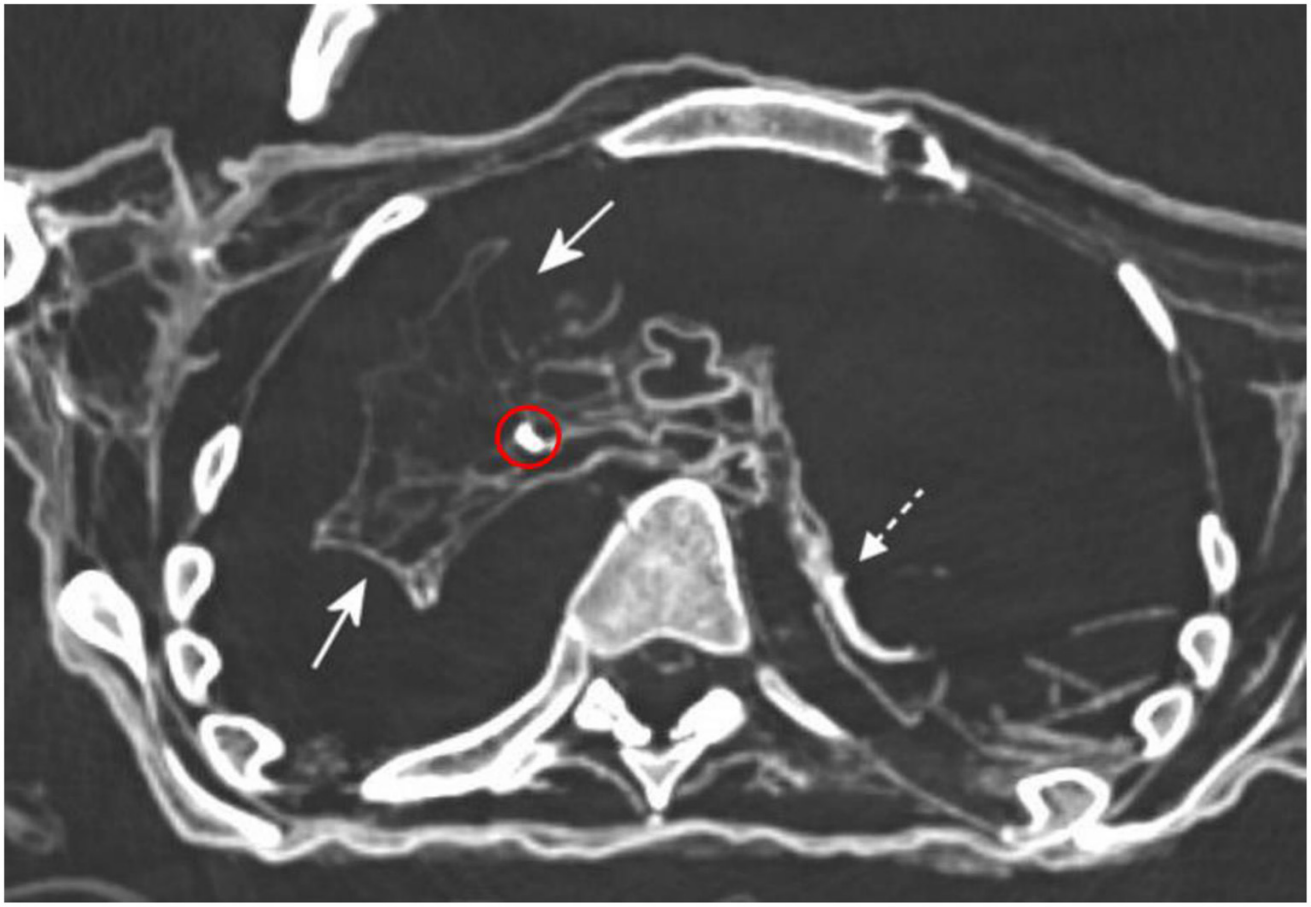
Axial CT reconstruction of the thorax. The left lung was collapsed and flat, condensed at the dorsal chest wall (dotted arrow) in contrast to the right lung, which was only partially collapsed, bridged the space (arrows) and contained several focal calcifications (circle) (images: S. Panzer).

**Figure 7:**
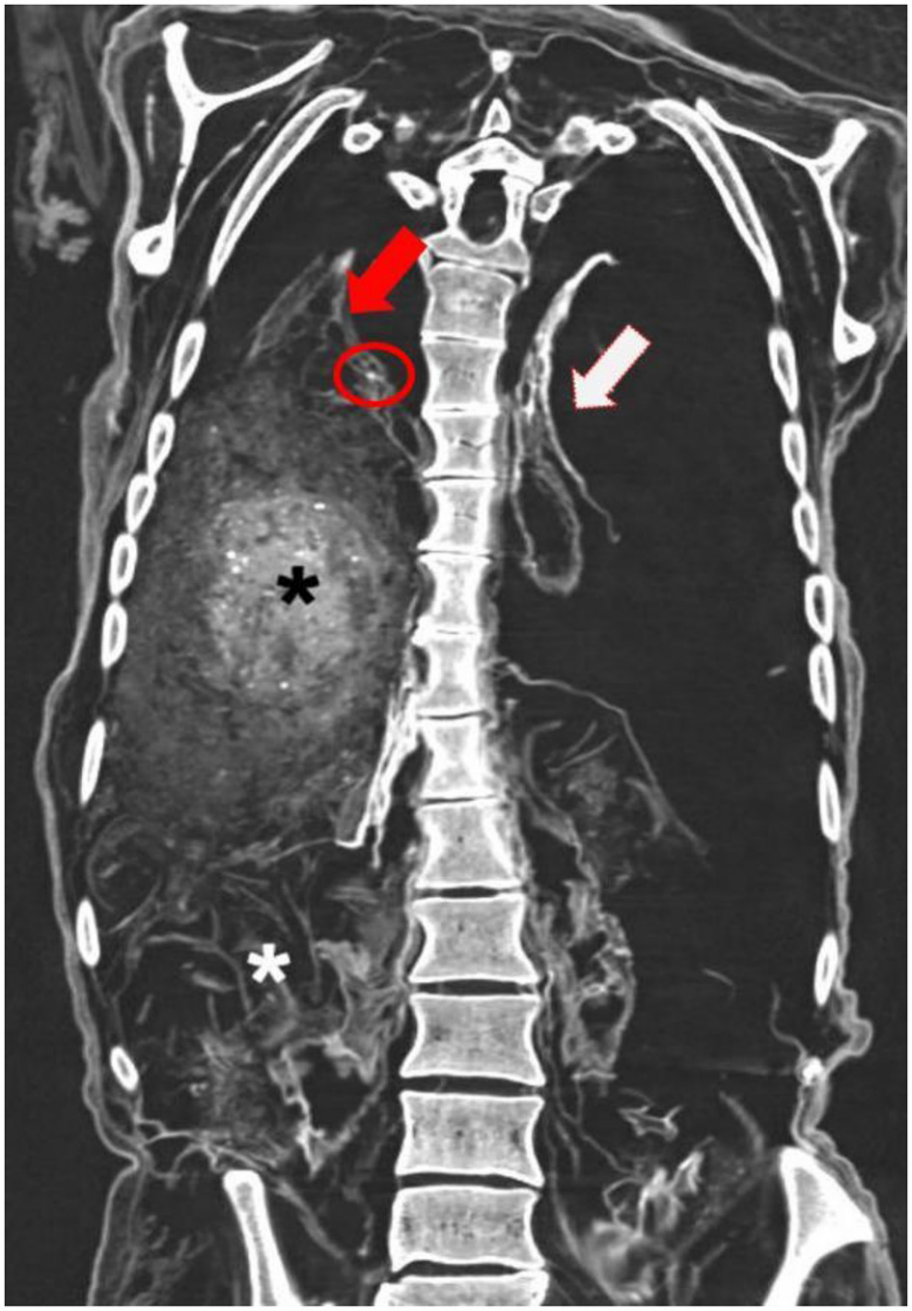
Coronal CT-reconstruction of the chest and abdomen of the mummy. The left lung (white arrow) was shrunken and flat, the right lung (red arrow) bridged the thoracic cavity. In the right lower lobe a small round cyst was associated with small spotty calcification (red circle). The abdomen was filled with foreign material (asterisks) of fine granular partly-formed material (black asterisk) and partly lamellar foreign material (white asterisk) (image: S. Panzer).

In the left thoracic cavity, the lung was typically collapsed and shrunken forming a flat plate along the dorsal chest wall. In contrast, the right lung appeared to span the thorax and was circumferentially attached to the ventral and dorsal chest wall as well as the diaphragm. Within the lung parenchyma several spots with round calcifications were detected both in the lung periphery and the center (Figure 6). Furthermore, in the basal right lower lobe a small round translucent cystic space was detected that contained at its margin a few spotty radiodense inclusions (see Figure 7).

Beyond this, most remarkably, major parts of the abdominal cavity, in particular on the right side were filled with heterogeneous foreign material. In contrast to the right side, the left side of the abdominal cavity contained far less foreign material. Furthermore, this filled the lower abdomen (and the pelvis), but did not extend until the left diaphragm. The foreign material consisted of either fine granular partly-formed material with tiny calcifications, or more compact lamellar sheets. The material filled the abdominal cavity reaching the intact right diaphragm. The various types of foreign material could not be further identified on the basis of the radio-morphology.

**Figure 8:**
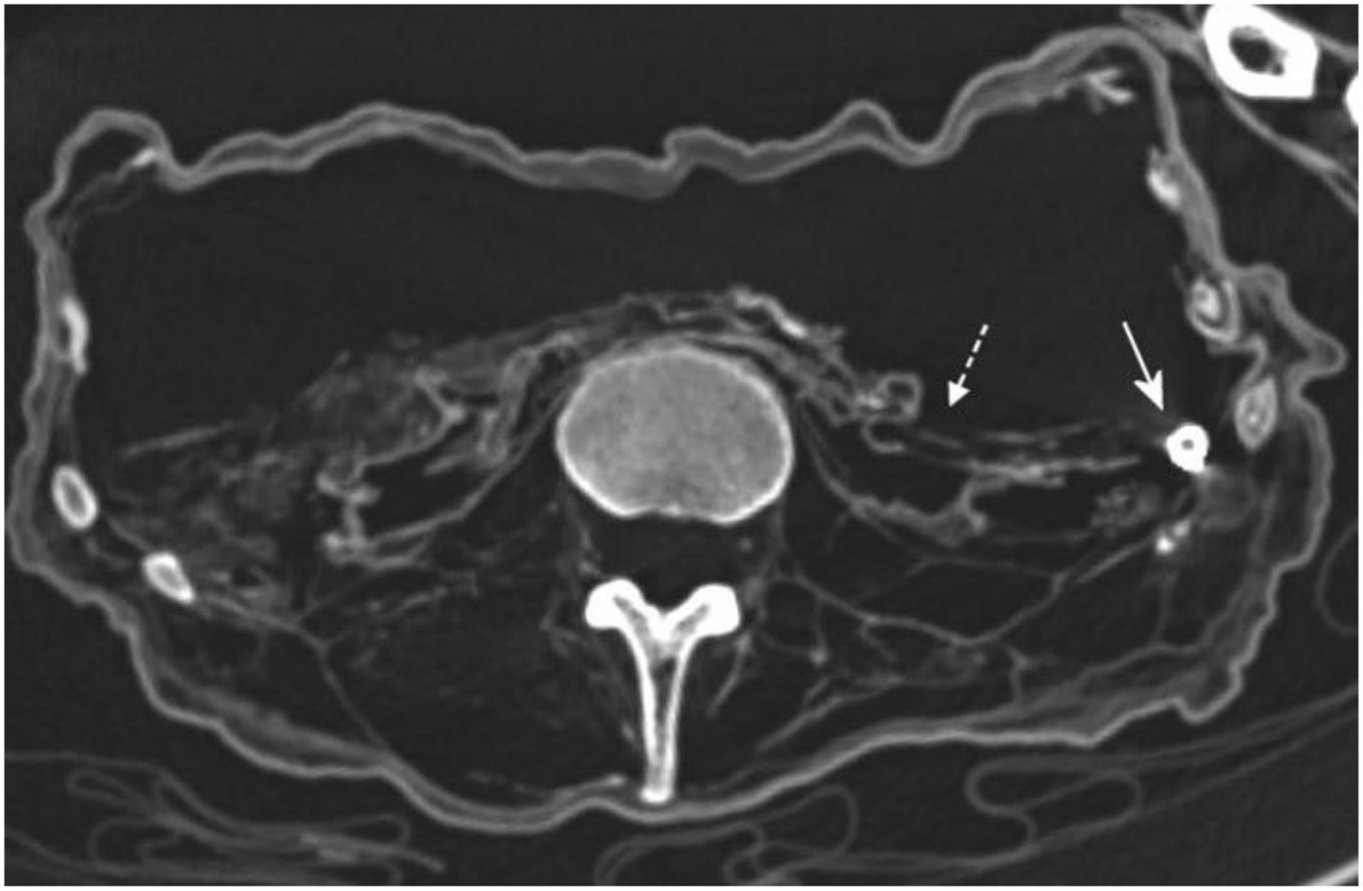
Axial CT reconstruction of the pelvis. On the left side the round foreign material was clearly seen (arrow) as a radiodense sphere with an empty center. This was embedded into lamellar foreign material and possible remnants of intestinal tissue which was seen on both sides of the pelvic cavity (dotted arrow) (image: S. Panzer).

Furthermore, the CT scans clearly identified the small roundish foreign body (about 1 cm in diameter) within the left pelvic cavity that had previously been seen on routine X-rays (therefore see also Figure 2). The CT additionally revealed that this foreign object had an empty center such as in a hollow sphere. The wall of the sphere was strongly radiodense. These images did not provide further identification of this foreign material (Figure 8).

### 3.4 Focal autopsy and procurement of the intra-abdominal foreign material

The most surprising finding was the presence of copious inhomogeneous foreign material filling the abdominal (and pelvic) cavity. Since the CT scans did not allow any further identification of this filler, the chest and abdominal cavity was opened through a dorsal paravertebral longitudinal incision of the body wall which was easily closed following sampling, in order to restore completely the outer integrity of the mummy. This permitted the removal of the foreign material for further analysis. Additionally, it provided access to the right lung for further analysis of the pathological changes.

**Figure 9:**
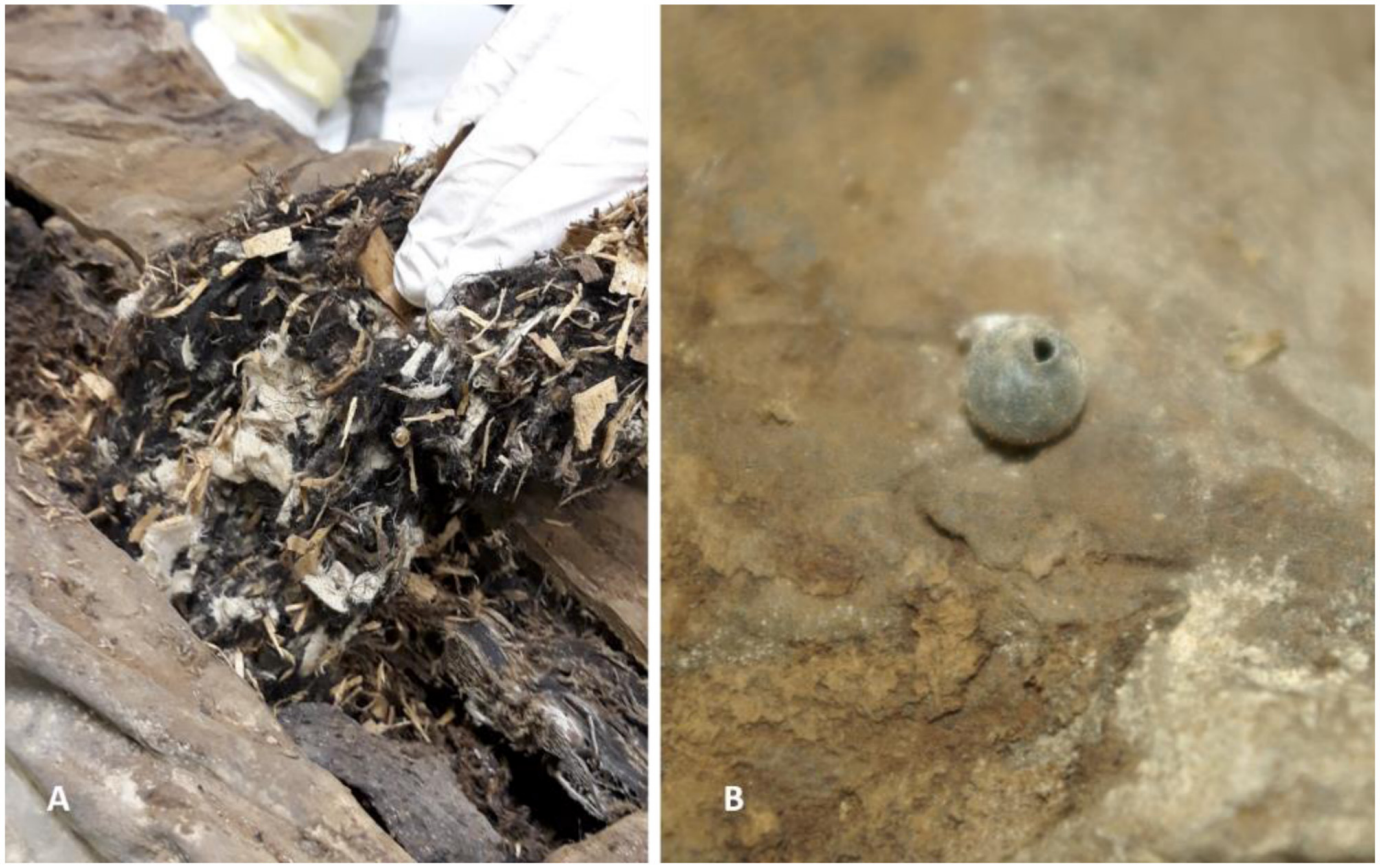
**(A)** Removal of parts of the foreign material from the dorsal abdominal wall revealed a mixture of fragmented white fabric, small wood chips and plant material along with some brownish amorphous tissue residues. **(B)** The round foreign sphere detected in the left pelvis had a small hole with a raised lip (images: A. Nerlich).

After opening the chest wall, numerous wood chips and small branches of wood were detected. Both were mixed with a coarse mud-like material, as well as many layers of fabric of varying quality and fabrication. The latter were severely degraded (Figure 9A) and occasionally contained few cloth accessories, such as buttons. Similarly, the round foreign body in the left pelvis was detected and removed. This was a small hollow sphere with two small holes with a slightly raised lip on opposite sides (Figure 9B).

**Figure 10:**
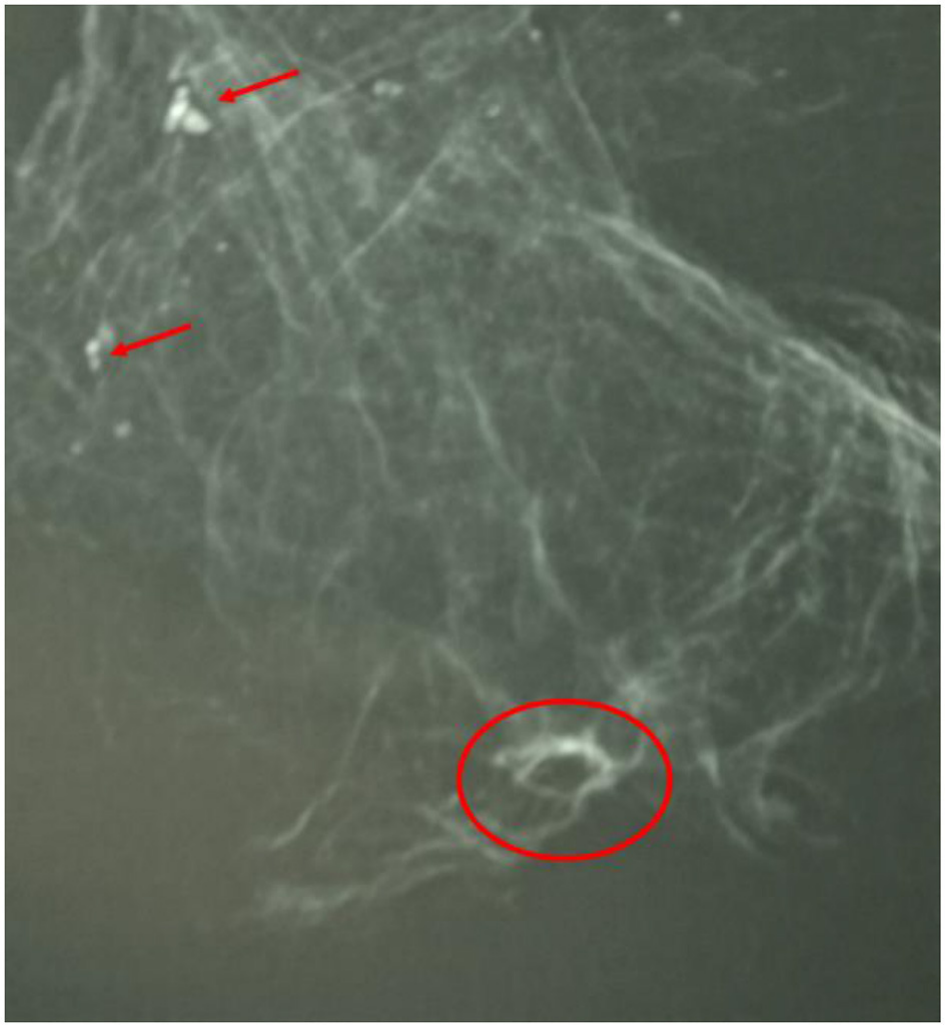
Contact X-ray of the removed right lung. This confirmed the focal calcifications (red arrows) and the cyst with capsular margins (red circle) (image: A. Nerlich).

Additionally, the complete right lung, which was partly fixed to the chest wall and the otherwise intact diaphragm, was removed. The lung lobe was brittle; its surface was irregular with remnants of focal adhesions to the chest wall. On contact X-ray, the already (by the CT-scans) identified radiodense inclusions (obviously calcifications) and the cyst could clearly be identified (Figure 10).

### 3.5 Isotope investigations

Further data came from the results of the stable isotope analyses on the type and composition of the diet, and radiocarbon dating (14C analysis), which allowed an estimate of the year of death.

**Figure 11:**
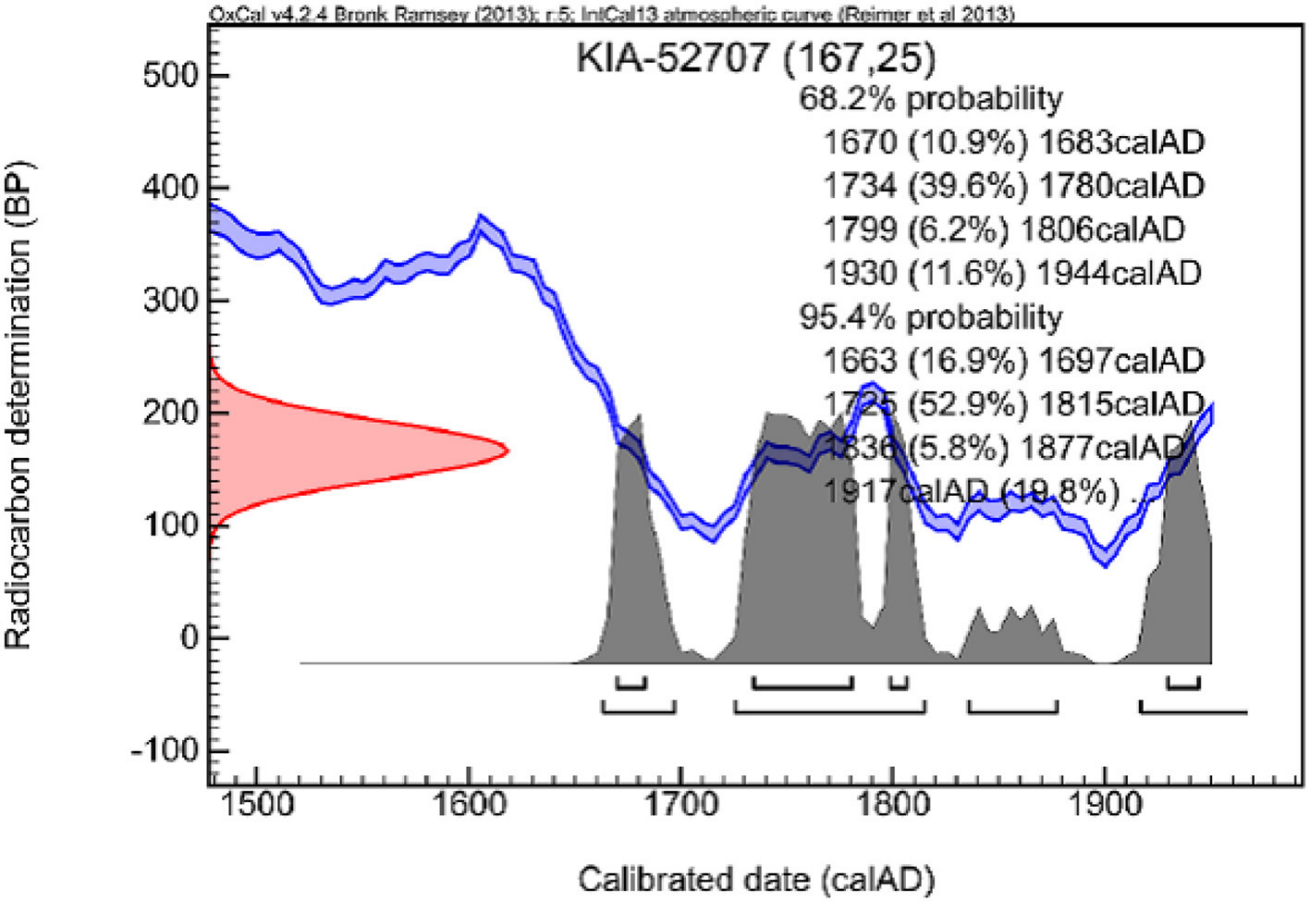
Radiocarbon dating of a piece of skin protein. The most probable date of death was between 1734 and 1780 AD (provided by Leibniz-Laboratory for Radiometric Dating and Isotope Research, Kiel University, with permission).

The radiocarbon dating of a skin sample yielded a radiocarbon age of 167 +/– 25 years, which, according to the calibration, occurred with a probability of 95.4% in several time periods between 1663 and 1944, with the period 1725–1815 having the highest probability for the time of the individuals' death (52.9 %) (Figure 11).

Stable isotope analysis of carbon (^13^C/^12^C), nitrogen (^15^N/^14^N) and sulfur (^34^S/^32^S) was carried out on three different tissue biopsies; collagen from dentine, which covers the period from childhood to adolescence, collagen from the vertebrae as an overall measure for the last 15–20 years, and finally on a skin and subcutaneous tissue sample that reflects the last 4–6 months of life. These different time periods can be used to identify a change in nutritional patterns during the person's life as well as a possible deterioration in health status before death.

The stable isotope signatures (Table 1) showed a high-quality diet with a comparatively high proportion of animal protein in his diet, which roughly corresponds to the diet of contemporary upper-class population groups (monastic and noble) (32–34). The patterns showed relatively constant values between childhood and adulthood, indicating a similar composition of food during his lifetime. He met his protein needs mainly through products from terrestrial animals and also from fish. The specific isotope signature of the skin sample indicated deterioration in his health in the last months of life.

**Table 1:** Stable isotope ratios in the collagen from various tissues of the mummy.

**Tissue type**	**Amount of collagen [%]**	**C/N [molar]**	***δ^13^C [‰]***	***δ^15^N [‰]***	***δ^34^S [‰]***
Dentin (tooth)	10.6	3.1	−19.22	13.70	6.12
Vertebral bone	16.5	3.1	−19.41	13.28	6.81
Skin	n.d.	3.5	−20.41^*^	15.32^*^	8.30

^*^Values for skin are corrected for bone collagen [−1.2 ‰ for δ^15^*N* and +0.2 ‰ for δ^13^C (56)].

### 3.6 Histological tissue analysis

Microscopical analysis was performed on a sample of skin tissue, a bone sample (vertebral bone) and several samples that had been obtained from the right lung. These latter were specifically taken from a central calcified spot and from the peripheral cystic space. The bone and the lung samples were carefully decalcified before embedding.

On histological slides the skin sample showed a complete loss of the epidermis as well as a lack of all nuclear structures, whereas the dermal collagen network was excellently preserved and appeared normal [data not shown]. Similarly, the compact and cancellous bone sample was normal in its histological structure excluding major generalized metabolic disorders affecting bone, such as in vitamin deficiency [data not shown].

**Figure 12:**
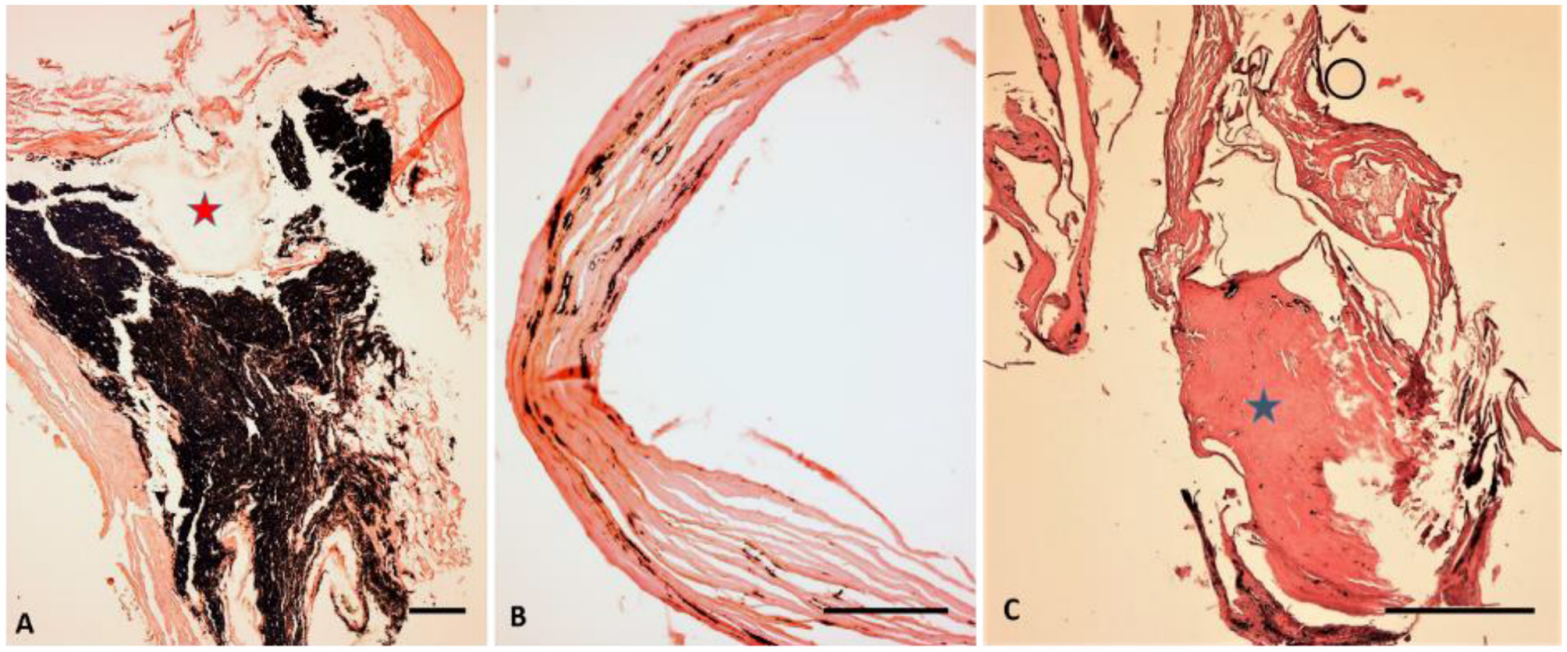
Histological investigation of lung tissue samples. **(A)** A presumed hilar lymph node showing major anthracosis (black pigment) and focal calcifications (asterisk). **(B)** The wall of the cyst is made of fibrous tissue with small inclusions of anthracosis pigment. **(C)** On another section a small heterologous ossification focus is seen (asterisk). (**A–C**: H&E staining, bar 100 μm, all samples decalcified) (images: A. Nerlich).

The central calcified lung node clearly showed the remnants of a hilar lymph node with dystrophic calcification and extensive anthracosis (Figure 12A). The findings are consistent with a tuberculous infection with secondary calcification. A section through the wall of the cyst revealed a fibrous pseudocapsule containing small inclusions of anthracosis pigment (Figure 12B) as well as small heterotopic ossifications (Figure 12C). This is well in accord with a tuberculous cavity as the result of a chronic florid tuberculous infection.

### 3.7 Toxicological analysis

In order to identify potentially poisonous elements, a toxicological analysis was performed. This covered 24 relevant elements; the results are presented in Table 2.

**Table 2:** Toxicological element analysis.

**Element**	**Mummy tissue [μg/kg]**	**Reference data [μg/kg]**
Aluminum	3.310	< 4.000
Antimony	80	n.d.
Arsenic	3.170	< 500
Beryllium	1.530	< 2.300
Cadmium	2.950	< 4.000
Cobalt	50	< 150
Gold	< 25	< 135
Indium	< 25	< 25
Copper	72.800	< 10.000
Molybdenum	200	< 300
Nickel	100	< 250
Palladium	80	< 25
Platinum	< 25	< 25
Mercury	530	< 500
Silver	< 25	< 25
Thallium	< 25	< 25
Uranium	40	< 25
Vanadium	< 25	< 25
Bismuth	< 25	< 25
Zinc	942.100	< 61.000
Tin	110	< 1.000
Zirconium	< 25	< 25

n.d., not detectable; reference data came from liver, lymph node or skin samples from recent material (21, 23) (see also text below).

A significant problem exists with the indicated reference values since present day analyses are undertaken on blood, urine or fresh tissue material. The effect of drying of the mummy tissue results in some unquantifiable concentration of the elements analyzed so that slightly elevated values in the mummy sample were disregarded. The given references mainly rely on measurements of liver, lymph node or skin samples from recent material (21, 23).

Taking these limitations into account, the interpretation of the data in Table 2 suggested the slight elevation of concentration in antimony, palladium, mercury, and uranium should be disregarded and classified as non-pathological. In contrast, the values for arsenic, copper and zinc were elevated while those of copper and zinc were markedly increased. With respect to arsenic this might have come from contamination during the preparation of copper and/or zinc so most probably representing a contamination and not an intentional preparation (35).

Further analysis of the sample by GC-MS did not show any positive pattern for psychoactive substances (including neurotoxins) or other poisons; the surrogate marker for alcohol consumption, ethyl glucuronide, was also negative.

### 3.8 Analysis of the foreign material

**Figure 13:**
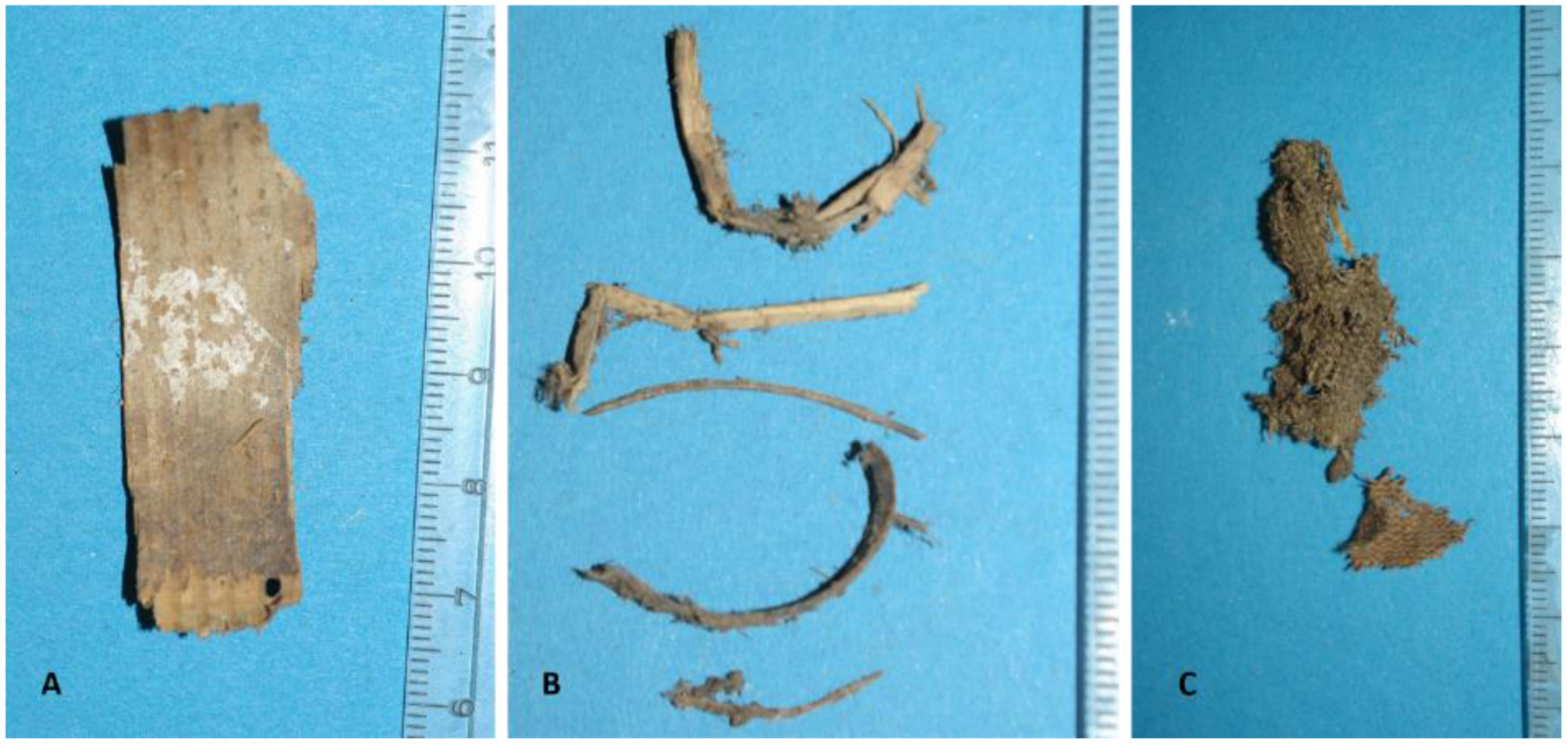
Analysis of the packing material obtained during the partial autopsy of the abdomen. **(A)** Typical wood chip. **(B)** Twigs of various plants. **(C)** Small fragment of a simple fabric made of hemp or flax (images: A. Nerlich).

In an attempt to identify further the material that had been used for filling the mummy's abdomen, part was subjected to macro- and microscopical analyses. The following foreign material was identified on the basis of its surface structure; the main bulk consisted of wooden chips from fir and spruce and severely fragmented pieces of wooden branches, these latter could not further be sub-classified. This mixture was intermingled with fragments of fabric of simple stem fibers, prepared in plain weave, mostly consisting of hemp and flax (Figure 13).

**Figure 14:**
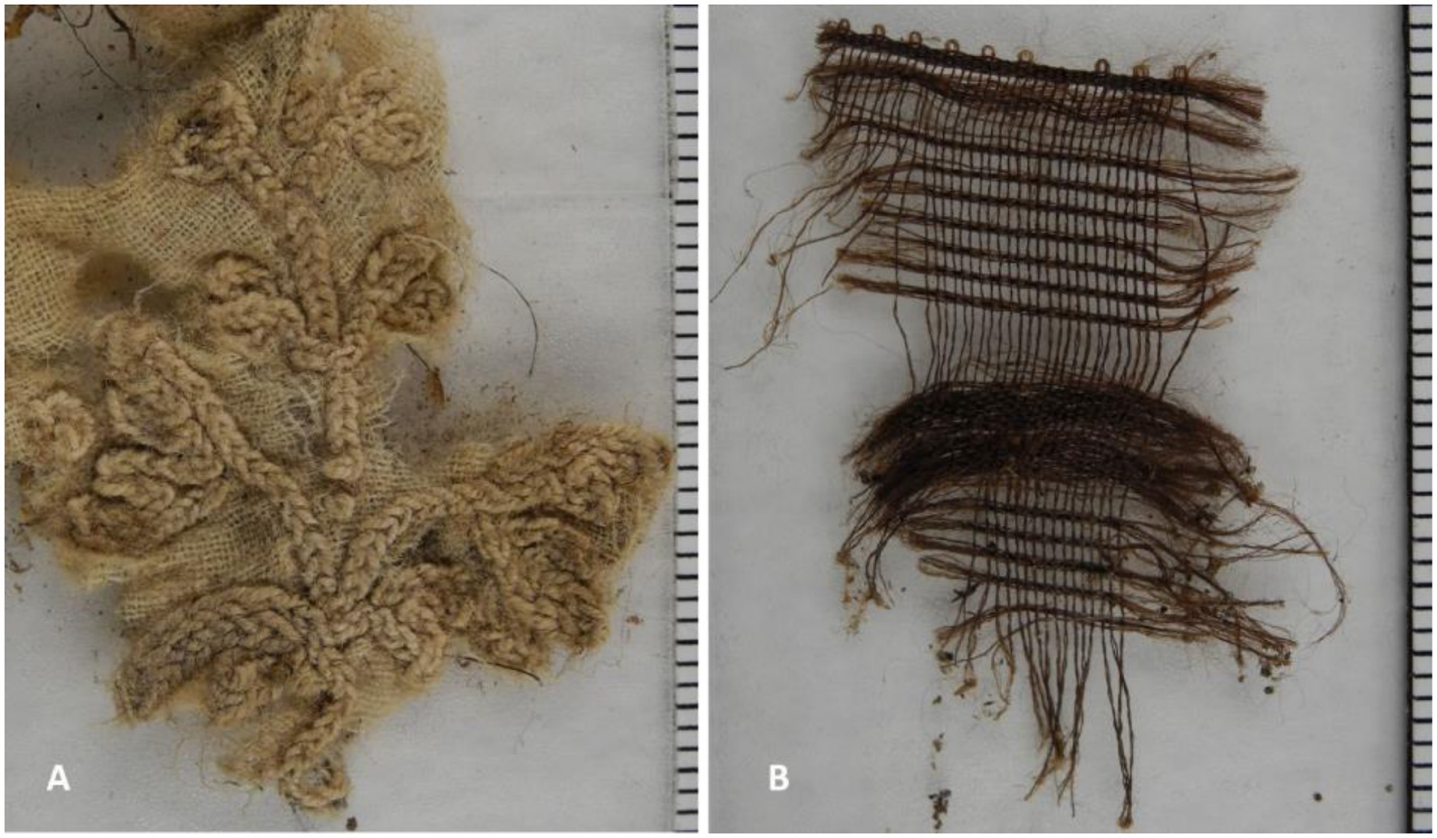
Further findings of special fabric tissue from the material detected in the mummy's abdominal cave: **(A)** A piece of cotton with an elaborate floral pattern. **(B)** Fragment of a silk fabric such as used for the mummy's cross (images: A. Nerlich).

A further major part of the packing material consisted of linen fabric for which use was not obvious. Besides some typical wooden buttons which had lost their textile coating, other types of fabric were occasionally identified. In particular, a piece of cotton in plain weave was found which was decorated with a crocheted floral ornament. One further particular piece of fabric was even identified to consist of expensive silk gauze in an elaborate pattern, such as seen to cover the cross which the mummy held in his fingers (Figure 14; for the cross see also Figure 3).

**Figure 15:**
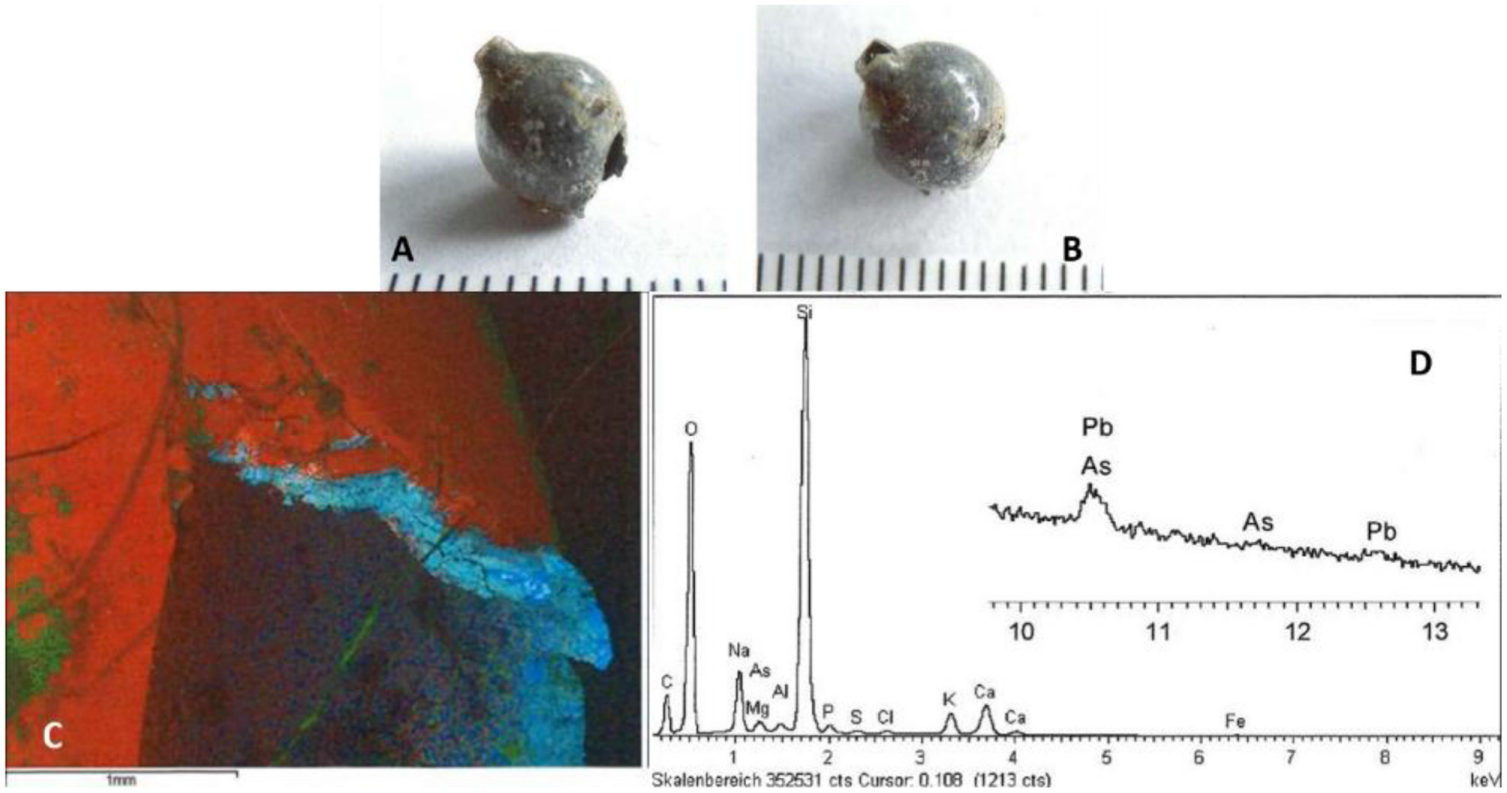
Analysis of the intrapelvic sphere: **(A, B)** Macroscopic aspects of the sphere showing a glistening surface and the two holes. **(C)** Scanning electron microscopy of the sphere's surface showing the silica-based glass sphere (blue) covered by a thin layer of lead and arsenic (red). **(D)** FITR-spectrum of the surface confirming the cover by lead (Pb) and arsenic (As) [images: **(A, B)** A. Nerlich; **(C, D)** K. Gonda, with permission].

Finally, we investigated the round hollow object that was found in the pelvic cavity. On external inspection, this object was spherical with a shiny surface and two holes opposed to each other. One of the holes seemed slightly damaged while the other one had a small raised circumferential bulge or lip to the edge. Subsequent scanning electron microscopy with Fourier-transform infrared spectroscopy (FITR) revealed a sphere made of typical glass (silica) that had been impregnated with a very thin layer of lead plus some arsenic on its surface; confirmed by a typical spectrum for these (Figure 15). Again, this metallised glass bead may have been used as decoration in embroidery, possibly monastic in origin or was part of a broken rosary.

## 4 Discussion

In 2003, Arthur Aufderheide reported in his fundamental book “The Scientific Study of Mummy Research” (1) on the “air-dried chaplain”, an adult human mummy. It was found in a small village of Upper Austria and was first scientifically described in 1967 by Kleiss (6) where the mummification process remained obscure. Even a more detailed scientific investigation, performed in the early years of 2000 by the Austrian pharmacologist Bernhard Mayer, did not uncover the mechanisms that had kept this human body in a fairly stable mummified condition for a long period of time. Nevertheless, the investigation with a portable X-ray machine detected a radiodense foreign object inside the body which raised the hypothesis that the historical individual had succumbed to the harmful ingestion of a poisoned capsule. These rumors in the local population culminated in a miraculous combination of “eternal conservation” (like in holy bodies of saints) and a ruthless attack against a pious man (8, 9, 11, 12).

Our present multidisciplinary study was undertaken to:

- Confirm the identification of the historic person as that of the presumed parish vicar Franz Xaver Sidler.
- Unravel his life and disease history as far as possible.
- Identify the most probable cause of death.
- Search for the reasons for the very good preservation of the mummified body over a long period of time.

### 4.1 Identification of the individual

The first documents reporting on an unidentified mummified body in the crypt of St. Thomas' church date back to 1849 (edited in 1850) (10). From 1866 a guidebook for tourists included the information that the mummy was supposed to be a priest (36).

Also rumors attributed the mummy to a local priest that had served as the parish vicar in the first half of the eighteenth century and who had died in St. Thomas am Blasenstein in the year 1746. This local aristocrat, named Franz Xaver Sidler von Rosenegg (sometimes also written as “Sydler”) had been born in 1709 in a small Upper Austrian village (Kreuzen) nearby St. Thomas as the 13th child to a civil servant who had close ties to the neighboring Augustine monastery of Waldhausen im Strudengau. In consequence Franz Xaver entered the monastery at a young age from where he was assigned to the parish of St. Thomas am Blasenstein around the year 1740. Usually, the assignment typically lasted about 10 years; according to parish records Franz Xaver Sidler died on the 3rd September 1746, before completing his term. He was only 37 years old. A letter announcing Sidler's death to the Abbot of the Benedictine monastery in Seitenstetten (Lower Austria) indicated that he was buried on the next day in St. Thomas (37). The documents, however, did not disclose any information about the cause of death nor does any further biographical information on him exist. Rather, the rumor concerning the “miraculous” mummy led to devotion, such that up to 1934 the parish celebrated a Mass dedicated to Sidler every year (11).

Our study identified the largely well preserved mummy of an adult male. Anthropological analyses suggested an age at death of between 30 and 50 years with the most plausible span between 35 and 45 years. Radiocarbon dating placed his period of death most likely between 1734 and 1780 CE. All these dates match Sidler's life. The stable isotope pattern indicated a high-quality diet based on Central European grain varieties and a large proportion of products from terrestrial food animals and possibly inland fish. Consumption of marine food was not apparent. This is well in line with the expected rural food supply of a local parish vicar and also matches the pattern seen in contemporary monastic and lower aristocratic populations from comparable South German regions (33, 34). Such a dietary pattern is also observed during his childhood, which is in accordance with his local aristocratic descent. The isotopic pattern of the last months of life was different and probably reflects reduced food availability due to the War of the Austrian Succession (which lasted until 1748) or final metabolic stress due to a severe wasting disease (38). Finally, the lack of any major stress signs in the skeleton, along with the absence of localized or generalized joint degeneration indicated an easy life without hard physical activity well in line with his position as a priest.

In summary, our multidisciplinary approach provided multiple sources of evidence to support the conclusion that the mummified body was that of Franz Xaver Sidler von Rosenegg (1709–1746).

### 4.2 Information about life, disease and death of the historical individual

Given that the mummy is that of Franz Xaver Sidler von Rosenegg and that, as a monk, he had fairly stress-free activities of daily living, and mostly adequate nutritional input, he had major periodontitis which is typically the result of insufficient oral hygiene as frequently noted in the skeletal remains of contemporaneous monks (39).

The mummy showed some evidence of chronic paranasal sinusoidal inflammation consistent with long-term pipe smoking. The dental findings of retraction of the chewing surface of several upper incisors are also consistent with this. The deposition of tiny coal particles in the lower airways that are usually transported to interalveolar septa and particularly accumulate in the hilar lymph nodes, both of which are seen in the lung histology here, suggest significant pulmonary anthracosis. Besides any possible smoking habit, the widespread use of open fires, especially for heating ovens in cold periods and cooking in earlier times, may have significantly contributed to this. This has also been seen in mummy lung tissue samples of a nineteenth century Bavarian aristocrat that has been recently investigated (15).

Anthracosis itself has no pathological consequences but the lung showed major pathology. On the right side multiple flecks of calcification were found in the periphery and the central (hilar) areas, highly suggestive of chronic pulmonary tuberculosis (15); the lung spanned the right thoracic cavity and was fixed to the pleura which is the end stage of major pleuritis that may result from chronic tuberculosis. This diagnosis was further confirmed by the histological evidence of local calcification in a hilar lymph node (which itself was identified by the significant anthracosis). The resulting pattern is that of a typical primary complex of lung tuberculosis which usually consists of a peripheral focus and an affected hilar lymph node (40). Whether this focus was one of the additional peripheral calcifications or the cystic structure in the right lower lobe remains unknown. The latter, as suggested on CT scans and localized by contact radiography following partial autopsy, showed, at least in part, a fibrous capsule (with minor anthracosis) and focal heterotopic ossification; a sign of a chronic and possibly still active process.

All this suggest a chronic, presumably still active, lung tuberculosis with typical primary complex formation and a peripheral cavity. The latter is well known to result in an acute pulmonary hemorrhage, particularly in chronic active inflammation, through the acute erosion of pulmonary vessels. Similar hemorrhage has been a frequent cause of death in non-treated cases of tuberculosis in the past (41, 42) and may also have been relevant here.

In this respect, we have to admit that we did not test for mycobacterial ancient DNA (aDNA) which has previously shown to provide positive results in both bone (43) and lung parenchyma (44) of ancient Egyptian mummies. A successful molecular detection, however, is dependent from the material and the stability of the aDNA (43). The aforementioned diagnostic criteria in radio- and histomorphology clearly are in favor of the diagnosis of chronic tuberculosis.

Also, the altered isotope pattern for the last months of his life is consistent with chronic, possibly active, tuberculous lung affection. Specifically, the increase in the nitrogen isotope ratio value suggests a physiological change in metabolism caused by an exhausting disease such as chronic active inflammation with damage to an important organ system or a malignant disease (38). Furthermore, the probable residues of a belt encircling his waist before mummification was evident and may indicate that he had lost a considerable amount of weight in the final period of his life.

There is no evidence that Franz Xaver Sidler suffered from other organs having tuberculosis (by haematogenous spread) and no signs of malignancy. Although a final tuberculous sepsis (so-called Landouzy sepsis) cannot be fully excluded. In total, we have good evidence that he died of acute severe pulmonary hemorrhage due to destruction of lung vessels by an ongoing infection associated with a tuberculous cavity although we have no data on the presence of mycobacteria (by detection of mycobacterial aDNA) in the lung/the presumed tuberculous caverna.

### 4.3 Measures for the preservation of the cadaver

The most surprising observation was that the abdominal and pelvic cavity was filled with considerable amounts of a mixed foreign material. Since both the ventral and dorsal body wall was completely intact this packing can only have been inserted via the anal canal. On careful inspection, the latter seems somewhat enlarged, but not excessively wide. It may speculate be that the anal canal was plugged after death in order to prevent the leakage of *post mortem* body fluid. However, at the time of all the inspections and investigations i.e., since the first scientific description by Kleiss in 1967 (7), the mummy was without clothes, and there was no report or remnant of any anal plug. It can therefore be concluded that the abdominal body cavity had been packed via the anal canal, and that any eventual dilatation of the anal canal was addressed before the body's burial.

If this assumption is correct, the access via the anal canal would have required an incision of the intestinal wall of the upper rectum or sigmoid colon to push the foreign material into the pelvic and abdominal cavity. Although this assumption is speculative this is the only way to insert the material. Interestingly, the right side of the abdomen is filled with more foreign material than the left one. One possible reason therefore may lie in the fact that the apico-caudal orientation of the insertion zone of the mesenterium “divides” the abdominal cavity into a “right” and a “left” side. So we may speculate that the embalmer incised not only the wall of the upper rectum or colon sigmoideum, but also the lower mesenterium and thereby opened the right abdominal side more easily to insert the material. In this scenario, the left side of the abdomen would have been filled by the convolute of the small intestine (which may further on have been destroyed in part by autolysis) so that the left abdominal cavity contains less of the foreign material. Nevertheless, we see foreign material in the left pelvic cavity and also minor material in the left abdominal cavity. In this respect, it is less plausible that the body might have been shifted first to its right side before it was brought into the final supine position. The aforementioned mesenterial insertion would have prevented a major dislocation from the left to the right side when turning the mummy. Accordingly, we have no indication for such a maneuver.

Besides this unusual filing, we identified the glass sphere as a glass pearl such as usually used e.g., in rosaries (45) since the fifteenth century. This is particularly supported by the molecular analysis of the sphere. In this respect, it is noteworthy that similar glass pearls were manufactured in near-by glass factories in the German-Bohemian-Austrian glass industry belt (45)—which is very near the village of St. Thomas am Blasenstein. It may be speculated that the glass bead incidentally came into the body during the filling procedure, either as part of a damaged rosary or as an application of an ecclesiastical ornate fabric or embroidery. Since only a single glass pearl was found, we can exclude that a—more or less complete—rosary should have been added to the filling material for religious reasons.

Finally, the wood chips, twigs (possibly pre-dried), and significant amounts of textile fragments would have resulted in rapid absorption of all decaying matter and blood fluids from inside the body. Interestingly, the toxicological analysis revealed excessively high values of zinc (with some copper that may have been contaminated with small amounts of arsenic). Zinc, and especially zinc chloride, are known to react strongly hygroscopically i.e., have a strong drying effect (46); likewise, zinc chloride has been used as an antimicrobial and disinfecting agent for a long time (47, 48). This may have been a further and important factor for the rapid stabilization and drying-out of the body from inside. Possibly the exterior may have been treated but it was not possible to find evidence to confirm this. However, the significantly worse preservation of the face (and feet) argues against simple external application of any zinc crystals. As an additional factor, the packing from the body cavity may have restored the shape of the abdomen and thereby prevented collapse of the ventral body wall.

To the best of our knowledge this is the first report on this type of conservation of a mummy, in particular the internal packing and chemical preparation via an anal route. All available embalming protocols rely on opening the cadaver, usually via an incision in the ventral abdominal wall, such as the widespread practice in ancient Egyptian mummification (5, 6). Possibly, the main reason therefore is that typical Central and Southern European burials are in cemeteries which results in prompt disintegration of the body when it comes into contact with soil. Accordingly, wooden chips, twigs, and fragments of fabric are no longer recognized as internal packing material. Furthermore, the detection of the material is only evident with more recently available radiological techniques, such as CT scans, or at autopsy of an intact body. This problem is highlighted in this case since the mobile routine X-ray in 2000 did not reveal the packing material, except for the small glass bead! In consequence, all previous X-ray studies of intact mummies do not exclude the packing technique used in this case.

While standard textbooks and articles do not report on this type of simple embalming [see e.g., (1, 2, 4)] there exist some written reports about simple methods of cadaveric conservation in the early eighteenth century England. While the embalming of socially high-ranked individuals for transport or prolonged laying-out of the dead body was not unusual, it was a highly costly practice performed in various European countries during the Middle Ages and the Renaissance period (49). At the beginning of the eighteenth century, in England, those carpenters who mostly also acted as undertakers expanded their business by offering a simple kind of preservation using “tar-and-sawdust embalming” (50–53). This usually comprised the external and occasionally internal application of sawdust; the latter sometimes described including the opening of the dead body, and the application of conservation material, such as tar and other materials. The most authentic description is given by a professional embalmer, the English surgeon Thomas Greenhill, whose income as an embalmer was obviously significantly affected by the new technique and who strongly condemned this new practice (54). Unfortunately Greenhill did not report on any specific technique and did not provide data about specific procedures and applied substances for the simple embalming. Further advice about similar procedures comes from an Italian embalmer (55) who described not only the external application of numerous wood chips for a short term conservation, but also described the use of a mixture of sawdust and zinc chloride with lavender (mixture: 50% volume sawdust +20% volume zinc chloride paste +1 % volume lavender). The method of internal application was not recorded in the brief description i.e., whether the anal canal or a ventral wall incision was used. Unfortunately, no reports are available from Austria or Central Europe, but it may be that this technique of simple preservation was transmitted orally.

## 5 Limitations of the study

Although we present an extensive study on the mummy from St. Thomas am Blasenstein, several limitations have to be mentioned: First, we did not perform a molecular analysis of the presumed tuberculous infection. Despite this, both the radio- and histomorphology are highly indicative for this disease. Therefore, we believe that the diagnosis—and ultimately the cause of death—in this case are correct. Furthermore, the toxicological analyses had to be compared with recent data which are available form fresh material. Although we took certain concentration effects by the drying of the mummy tissue into account, there are no direct reference values from other mummified corpses available. This problem may possibly be overcome in future when further naturally mummified bodies from comparable settings have been investigated. Then, the results of this study may be re-evaluated. Finally, we have no information on any (e.g., “chemical”) manipulation of the corpse' surface, especially during the first decades after death. This was also the reason why we did not analyse tissue samples from the body surface during our toxicological investigations, although the histological analysis of the mummy surface did not present any evidence for preservation techniques of the skin.

## 6 Final remarks

In summary, this extensive multidisciplinary approach not only confirmed the identity of the mummy, but also the reason for the good preservation of the dead body over a period of around 240 years in the Middle European climate. The evidence suggests that the preservation was performed to avoid the spread of infection by miasma. Possible later opening of the coffin or relocation of the human remains would have found a remarkably intact corpse and could easily result in miraculous beliefs by the local population. Needless-to-say future investigations of crypt burials should take note of this unusual type of embalming when undertaking planned analyses of human remains.

## Data Availability

The original contributions presented in the study are included in the article/supplementary material, further inquiries can be directed to the corresponding author.
